# Outcomes of children aged 6–59 months with severe acute malnutrition at the GADO Outpatient Therapeutic Center in Cameroon

**DOI:** 10.1186/s13104-018-3177-0

**Published:** 2018-01-24

**Authors:** Judwin Alieh Ndzo, Alan Jackson

**Affiliations:** 1 Faculty of Medicine, University of Southampton, Flat 3, 10 Cracknore Road, Southampton, Hampshire SO15 1JD UK; 2 Human Development and Health, University of Southampton, Southampton, UK

**Keywords:** Severe acute malnutrition, Outpatient treatment center, Treatment outcomes, Cameroon

## Abstract

**Objective:**

We aimed to assess outcomes [rates of recovery, default, case fatality; rate of weight gain and rate of Mean Upper Arm Circumference (MUAC) gain] of children aged 6–59 months with severe acute malnutrition (SAM) at the Outpatient Therapeutic Center at Gado Refugee Camp, Cameroon, in relation to international standards. We retrospectively analysed files of 254 children with SAM aged 6–59 months admitted from April 2015 to August 2016.

**Results:**

72.8% got discharged as recovered, 0.8% died and none defaulted. 26.8% got referred to stabilization center, mostly for poor weight gain (44.1%). Mean rate of weight gain was 4.4 g/kg/day and MUAC gain 0.3 mm/cm/day; median duration of treatment 44.5 days. Amongst those with marasmus, kwashiorkor and marasmic kwashiorkor, median duration of stay was 48, 24.5 and 36.3 days (p = 0.002); recovery rates were similar 73, 71.4, 71.4% respectively (p = 0.7); Median rates of weight gain, 4.4, 6.7 and 8.1 g/kg/day (p = 0.05). 49 children had been incorrectly diagnosed and treated as SAM. International Standards were met in terms of case fatality rate and default rate but not rates of recovery and weight gain. Separate gender charts must be used to calculate weight for height z scores as combined charts cause significant errors.

**Electronic supplementary material:**

The online version of this article (10.1186/s13104-018-3177-0) contains supplementary material, which is available to authorized users.

## Introduction

Nearly 20 million children under 5 years suffer from severe acute malnutrition (SAM) [1], mostly in Sub-Saharan Africa and South Asia [1–3]. SAM increases childhood mortality [1] and could lead to poor cognitive development [4–6]. The rising number of humanitarian crises has been accompanied by an increase in children suffering from malnutrition [7].

Initial guidelines for management of SAM advocated for inpatient management of all cases, but required skilled health personnel and expensive treatments [8, 9]. This was not feasible in settings of high prevalence of SAM where populations were poor [10, 11]. With time, health agencies shifted management of uncomplicated SAM to outpatient settings [12, 13]. In 2007, World Health Organisation revised its guidelines, adopting this practice [14]. Sphere and Valid International have produced guidelines which have been adopted by many countries [15, 16]. These state that successful OTCs should have CFR < 10%, Recovery rate > 75%, Default rate < 15% and rate of weight gain ≥ 5 g/kg/day [15].

The politico-religious crisis in Central African Republic has led to a high influx of refugees into neighbouring Cameroon mainly through Garoua-Boulai, a border town. The Gado Refugee Camp, situated about 20 km away from Garoua-Boulai, was set up by the United Nations High Commission for Refugees [17]. It had a population of about 30,000 refugees by 2016, 20% of whom were children less than 5 years [Outpatient Therapeutic Center (OTC) data]. The Gado OTC was within the Gado Health Center which was located 100 m from the camp. Both were run free of charge by the French Red Cross. The OTC covered over 90% of the target population.

This study aimed to retrospectively assess the outcomes [Rates of weight gain (RWG), rates of recovery, default rate and case fatality rate (CFR)] for a cohort of children aged 6–59 months diagnosed with uncomplicated SAM and followed up at the Gado OTC from April 2015 to August 2016, in relation to Sphere guidelines. This will add to existing evidence in the management of SAM and identify shortcomings in treatment that can be addressed.

## Main text

### Methods

#### Study design and population

Data were collected by the main researcher from files stored at the French Red Cross office at Garoua-Boulai. The OTC was run by 4 nurses trained by UNICEF. Combined gender growth charts were used to calculate weight for height z-score (WHZ). A physician at the nearby health center was assigned to review difficult cases. Active screening was done at the camp and health center by over 200 community volunteers and those meeting criteria for diagnosis of uncomplicated SAM [defined as WHZ score < − 3 [1] or Mean Upper Arm Circumference (MUAC) < 115 mm [1] or bilateral pitting oedema [1] and no medical complications were admitted at the OTC while those with complications (as defined by the Cameroon Protocol [18])] were referred to stabilisation center.

At each child`s visit, data (height, weight, MUAC, results of an appetite test [18]) were recorded in a coded file. A weekly ration of PlumpyNut was given (a lipid-based, multivitamin/mineral enriched, ready-made paste providing 170 kcal/kg/day [18]) and children asked not to eat household meals. Mothers were asked to report clinical symptoms in the last few days. Fever was defined as ≥ 38.5 °C and recorded using mercury thermometers. Malaria test was done using a Rapid Diagnostic Test (Histidine-Rich Protein 2) specific to *Plasmodium falciparum*. Antibiotics, vitamin A, antihelminthics and vaccines were administered according to the procotol. United Nations Children’s Emergency Fund (UNICEF) MUAC tapes were used to measure MUAC and locally made stadiometers were used, both with a precision of 0.1 cm; Uniscale scales for weight (100 g-precision).

#### Sampling

**Fig. 1:**
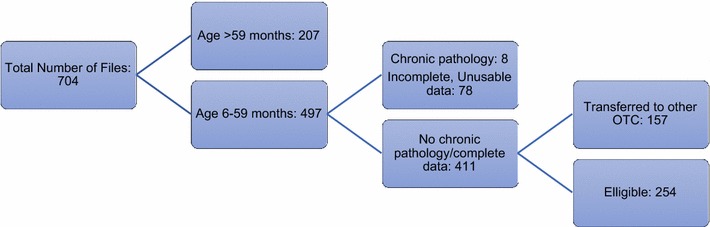
Sampling of study participants

704 files were found in the database admitted from April 2015 to August 2016 (Fig. 1). Ages of patients ranged from 6 months to 75 years. We included all 254 that met our inclusion criteria (new admissions aged 6–59 months with uncomplicated SAM [18]) and had complete, usable anthropometric information from admission to our desired outcome.

UNICEF/WHO guidelines are adapted for kids 6–59 months old, so assessing their applicability could only be valid for this age group. As outcomes were being assessed, those meeting criteria but transferred to another OTC at some point during follow-up could not be assessed as their outcomes were unknown.

#### Data collection and study variables

Raw data (demographic, clinical and anthropometric characteristics on admission, during follow-up and at discharge) were collected using google forms, then exported to Excel version 2016 for editing. Gender and age-specific WHZ, weight for age Z and height for age z were re-calculated using WHO Anthro version 3.2.2, which is based on WHO 2006 child growth standards [19]. IBM SPSS version 23 was used for data analysis.

We assessed mean age, wasting, clinical symptoms on admission, type of malnutrition [Marasmus (WHZ < − 3 or MUAC < 115 mm), Kwashiokor (oedema only but MUAC ≥ 115 mm and WHZ ≥ − 3) and Marasmic Kwashiokor (WHZ < − 3/MUAC < 115 mm and oedema) [1]]. Outcome variables were treatment duration, rates of weight gain, rates of MUAC gain, type of discharge defined as cured/recovered (Achieved target anthropometry for two consecutive weeks), death, referral to SC if seen to have any complications or defaulted (absent for two consecutive weeks).

These were compared to Sphere Standards [15].

Duration of stay was calculated in days from date of admission to date of exit. Rates of weight gain (RWG) (g/kg/day) was calculated using the formulae below [20]:

1 $$\frac{Weight \,on \,admission \,(or \,at\, no\, oedema) - Weight\, on \,discharge \,(g)}{Total \,duration\, of\, stay\, in\, days*Weight\, on \,admission\, in\, kg}$$

#### Equation 1: calculation of RWG for children

For those with oedema, admission weight was taken as the lowest weight (at no oedema) [20]. For RMG (mm/cm/day), it was logical to substitute MUAC in the formulae above. RMG and RWG were not calculated for children with OM who had not lost their oedema during follow-up.

#### Data analysis

Descriptives obtained for categorical variables included the variable count and percentages. For continuous variables, we obtained mean and standard deviation for normally distributed variables and median and Interquartile (IQR) for skewed ones. All outcomes were described with their probability (p) values and 95% confidence intervals (CI). The threshold for a type 1 error was set at 5%, statistical power at 80%. All statistical tests were 2-sided.

Pearson Chi square and Fischer’s test were used to investigate associations between categorical variables, and the independent t test and one-way ANOVA test were used to compare the mean of continuous outcome variables respectively across 2 and 3 level categories of explanatory variables. Mann–Whitney U and Kruskal–Wallis tests were used for skewed variables. All assumptions of tests were taken into account.

### Results

#### Validity of data

The OTC did not record admission WHZ for 29 of the 254 cases though weight and height had been recorded for these. As the OTC used combined gender growth charts, z-scores calculated by the OTC differed significantly from those calculated by authors using the same values for weight and height. The difference in values obtained by the OTC and the re-calculated values was highly statistically significant [p < 0.001, OR 0.1 CI (0.02–0.74)]. Z-scores presented in the results are those recalculated by authors.

#### Characteristics of sample on admission

From Table 1, 48% of the population were male and 52% female. The overall median age was 14 (IQR 6–59) months while mean WHZ was − 3.5 ± 0.8 for males, − 2.8 ± 0.9 for females (p < 0.001, CI 0.5–0.9); the mean overall MUAC was 116.8 ± 9.2 mm; 21 (8.3%) had oedema. Mean weight for age z-score was − 4 ± 0.9 for males and − 3.4 ± 1.0 for females (p < 0.001, CI 0.3–0.8); mean height for age z-score was − 3.0 ± 1.7 for males and − 2.5 ± 1.6 for females (p = 0.2 CI 0.1–0.8).

**Table 1:** Measurements at admission and discharge by categories of outcome

	Total (N = 254) N (%)/mean (SD/IQR)	Recovered, N = 185	Referred to SC, N = 68	Died, N = 1	Defaulted, N = 0
Admission variables					
Gender, N (%)					
Male	122 (48)	92 (75.4)	29 (23.8)	1 (0.8)	0.0 (0.0)
Female	132 (52)	93 (70.5)	39 (29.5)	0 (0.0)	0.0 (0.0)
Vomiting					
Yes, N (%)	62 (24.4)	45 (72.5)	16 (25.8)	1 (1.6)	0.0 (0.0)
No, N (%)	192 (75.6)	139 (72.4)	52 (27.1)	0.0 (0.0)	0.0 (0.0)
Fever					
Yes, N (%)	61 (24.0)	42 (68.9)	19 (31.3)	1 (1.6)	0.0 (0.0)
No, N (%)	193 (76.0)	143 (74.1)	49 (25.4)	0.0 (0.0)	0.0 (0.0)
Cough					
Yes, N (%)	66 (26)	50 (75.8)	15 (22.7)	1 (1.5)	0.0 (0.0)
No, N (%)	188 (74)	135 (71.8)	53 (28.2)	0.0 (0.0)	0.0 (0.0)
Malaria					
Yes, N (%)	50 (19.7)	39 (78.0)	10 (20.0)	1 (2.0)	0.0 (0.0)
No, N (%)	204 (80.3)	146 (71.6)	58 (28.4)	0.0 (0.0)	0.0 (0.0)
Diarrhoea					
Yes, N (%)	90 (35.4)	70 (77.8)	20 (22.2)	1 (1.1)	0.0 (0.0)
No, N (%)	164 (64.6)	115 (70.1)	48 (29.3)	0.0 (0.0)	0.0 (0.0)
Age, months					
Median (IQR)	14 (9–24)	14 (9–24)	14 (10–24)	36.0	–
WAZ					
Median (IQR)	− 3.7 (1.0)	− 3.8 (− 4.3 to − 3.1)	− 3.6 (− 4.2 to − 3.0)	− 4.0	–
WHZ					
Median (IQR)	− 3.1 (0.9)	− 3.1 (− 3.6 to − 2.7)	− 3.1 (− 3.6 to − 2.6)	− 2.4	–
HAZ					
Median (IQR)	− 2.8 (1.7)	− 2.9 (− 3.9 to − 1.6)	− 2.5 (− 3.6 to − 1.5)	− 4.2	–
MUAC					
Median, (IQR)	116.8 (9.2)	116.5 (112–123)	117.3 (112–123)	126.0	–
Discharge variables					
WAZ					
Median (IQR)	− 2.4 (1.1)	− 2.2 (− 2.8 to − 1.5)	− 3.1 (− 3.9 to − 2.2)	− 4.0	–
WHZ					
Median (IQR)	− 1.1 (1.3)	− 0.8 (− 1.2 to − 0.4)	− 2.4 (− 3.1 to − 1.7)	− 2.4	–
MUAC					
Median (IQR)	129.5 (10.3)	132.3 (127–138.5)	121.9 (114–128.8)	126.0	–
Duration of stay/days					
Median (IQR)	47.1 (24.9)	50 (33–71)	26.5 (10–55)	3	–
RWG^a^					
Median (SD or IQR)	4.4 (4.4)	5.6 (2.8–7.2)	1.3 (0.0–3.2)	0.0	–
RMG^b^					
Median (IQR)	0.3 (0.3)	0.3 (0.2–0.5)	0.1 (0.0–0.2)	0.0	–

^a^g/kg/day

^b^mm/cm/day

From Table 2, 184 (72.4%) had marasmus, and 63.25 of these were male. 14 (5.5%) had kwashiorkor, 64.3% of whom were female (64.3%); 7 (2.8%) had marasmic kwashiorkor, mostly males (85.7%); 49 (19.3%) had been misdiagnosed, did not have SAM and were incorrectly being treated as severely malnourished. Almost all of these were female (98%).

**Table 2:** Admission and discharge characteristics by type of malnutrition

	Marasmus, N = 184 N (%)	Kwashiokor, N = 14 N (%)	Marasmic Kwashiokor, N = 7 N (%)	p values	No SAM, N = 49 N (%) or Median (IQR)
Gender					
Male, N = 122	110 (63.2)	5 (35.7)	6 (85.7)	–	1 (2)
Female, N = 132	74 (36.8)	9 (64.3)	1 (14.3)		48 (98)
Age					
Median (IQR)	17 (9–23.3)	24 (22.5–32)	13 (9.1–21)	0.009	16.8 (12–24)
Duration of treatment					
Median (IQR)	48 (33.1–71.5)	24.5 (12–37)	36.3 (9.8–77.5)	0.002	35.5 (14.9–63.3)
RWG					
Median (IQR)	4.4 (2.2–6.8)	6.7 (0–10.6)	8.1 (2.0–13.6)	0.05	3.5 (1.8–5.3)
RMG					
Median (IQR)	0.3 (0.1–0.5)	0.3 (0.0–0.4)	0.5 (0.1–1.1)	0.06	0.2 (0.1–0.4)
Type of discharge					
Recovered	136 (73.9)	10 (71.4)	5 (71.4)		34 (69.4)
Referred to SC	48 (26.1)	3 (21.4)	2 (28.6)	0.7	15 (30.6)
Died	0 (0.0)	1 (7.1)	0 (0.0)		0 (0.0)
Defaulted	0 (0.0)	0 (0.0)	0 (0.0)		0 (0.0)

Diarrhoea was the most common symptom on admission (35.4%) followed by cough (26%), vomiting (24.4%), fever (24%). Prevalence of malaria was 19.7% on admission. Those with fever had highest rates of referral to stabilization care (31.3%). (Table 1).

#### Discharge characteristics of sample

72.8% were discharged as completely recovered; none had defaulted; 1 had died (0.8%). 26.8% were referred to SC after developing complications [poor weight gain (44.1%, anorexia (26.5%), infections (26.5%), persistent oedema (2.9%)]. Mean time to discharge was 47.1 ± 24.9 days. (Table 1).

Mean overall rates of weight gain and MUAC gain were 4.4 ± 4.4 g/kg/day and 0.3 ± 0.3 mm/cm/day respectively (Table 1). For those who had recovered, rate of weight gain was 5.6 g/kg/day.

Comparisons were made between children discharged as cured and those referred to the stabilization center, and while there was no significant difference in age, WHZ, height and weight for age z-scores, those who were cured had longer duration of treatment [50 days versus 26.5 days (p < 0.001, CI − 23.6 to − 10.5)], rate of weight gain [5.6 versus 1.3 g/kg/day (p < 0.001, CI − 5.4 to − 3.2)] and rate of MUAC gain [0.3 versus 0.1 mm/cm/day (p < 0.001, CI − 0.3 to − 0.2)] (Table 1).

Median duration of stay was 48, 36.3 and 24.5 days for those with marasmus, marasmic kwashiorkor and kwashiorkor respectively. There was no statistical difference in median rates of weight and MUAC gain and rate of recovery, in all three groups. (Table 2).

### Discussion

This is one of a few studies that focus on children with SAM as outpatients in an emergency context. Given the short time frame for data collection, a retrospective cohort was the best study design. It confirms that low mortality rates can be achieved in OTCs when guidelines are implemented with well-trained staff. It nonetheless highlights pitfalls in diagnosing and managing children with SAM at a highly funded OTC in an emergency context.

OTP did not meet Sphere standards in terms of recovery rate (72.8% compared to 75%) and RWG (4.4 g/kg/day compared to 5 g/kg/day) [15]. However, mortality (0.8%, less than the recommended < 10%) and default rates (0%, less than the recommended < 15%) were within sphere standards [15].

The lower rate of recovery could be because of the low threshold for referral to SC as free transportation, feeding and lodging were provided for the mother and other siblings. This probably accounted for low CFR. There was also good community follow-up by over 200 volunteers which accounted for 0% default.

Average overall rate of weight gain was slightly below Sphere standards but above for those who had recovered. Failure to gain adequate weight was the most common reason for referral. This raises questions on the use of RUTF, a high calorie meal providing up to 170 kcal/kg/day which, if properly consumed by the child, should result in RWG > 8 g/kg/day [18]. This indicates the mothers could be sharing the PPN with siblings or selling them.

The longer duration of stay for those with marasmus is not surprising as these were mostly males with very low WHZ on admission. Their comparatively low RWG during follow-up signifies that the more wasted children have lower catch-up growth than those with OM. A similar study [20] found a similar RWG for marasmic children but compared to those with OM in their sample, marasmic children had higher RWG.

The significant difference in nutritional status on admission between males and females could be explained by the fact that the OTC used combined gender charts which erroneously classify females with normal or moderate WHZ scores as severely malnourished. This explains why 49 children in our sample were admitted though they did not meet any criteria for SAM.

By admitting children with a fever (most of who ended up being referred to SC), the OTC did not adhere to the Cameroon guidelines.

### Conclusions

Severe acute malnutrition can be feasibly managed in OTCs when structures are properly organized. However, strict adherence to guidelines is necessary to achieve better outcomes. Gender combined growth charts should not be used in making a diagnosis of SAM as they increase potential for misdiagnosis. The Cameroon protocol should be revised, taking this into consideration. The OTC needs to improve on data management. Qualitative research should be done focusing on the use of OTC at home, to understand why despite being given adequate amounts, weight gain is stipp suboptimal.

## Limitations

Some files had incomplete data and were unusable, increasing potential for selection bias. Age was estimated by comparing birth timing with important festivities and events as birth certificates were not available; seasonal variations of prevalence of SAM were not obtained. For children with oedema, we assumed that the lowest weight recorded was the period of oedema loss.

## Additional files

**Additional file 1.** Complications of SAM warranting referral to Stabilisation Center. Describes various medical complications that would warrant admission to a stabilization center, or referral in the course of treatment at the outpatient treatment center.

**Additional file 2.** Comparing OTP Outcomes with Sphere Standards. Compares various outcomes of Gado OTP with respect to Sphere standards.

**Additional file 3.** DATASET SAM. Raw Data Set.

## Abbreviations

CMAM: Community-based Management of SAM
IQR: interquartile range
MUAC: Mean Upper Arm Circumference
OTC: Outpatient Therapeutic Center
SAM: severe acute malnutrition
UNICEF: United Nations Children`s Emergency Fund
WHO: World Health Organisation
WHZ: weight for height z scores
