# Supplementary material for: Outcomes of children aged 6–59 months with severe acute malnutrition at the GADO Outpatient Therapeutic Center in Cameroon
Source: BMC Res Notes. 2018 Jan 24;11:68. doi: 10.1186/s13104-018-3177-0 (PMC5782382; doi:10.1186/s13104-018-3177-0)
Supplement: Supplementary file 1 — Additional file 1. Complications of SAM warranting referral to Stabilisation Center. Describes various medical complications that would warrant admission to a stabilization center, or referral in the course of treatment at the outpatient treatment center. [file 13104_2018_3177_MOESM1_ESM.docx]

S1

| Complication | Description |
| --- | --- |
| **Failed appetite test** | Failed to complete a precise amount of RUTF in an hour (amount given per body weight) |
| **Generalised Oedema** | Oedema up to thighs, forearms and face |
| **Oedema + WHZ<-3** | Marasmic-kwashiokor |
| **Medical illness eg peneumonia, severe malaria, meningitis, dehydration, severe anemia, unconsciousness, hyper/hypothermia etc** | Only Rapid Diagnostic Test (RDT) was available for malaria; other diagnosis were clinical. Mother had to report symptoms over previous days eg vomiting. |
| **Oropharyngeal candidiasis** | By clinical observation |
| **Open skin lesions** | Clinical obersvation |
| **Static weight** | After 21 days |
| **Continuous weight loss for non-OM** | After 14 consecutive days |
| **Failure of oedema to start resolving** | 14 days |
| **Loss of ≥5% body weight for non—OM** | Any time |
| **Weight loss for two consecutive visits** | Any time |
