# Supplementary material for: Outcomes of children aged 6–59 months with severe acute malnutrition at the GADO Outpatient Therapeutic Center in Cameroon
Source: BMC Res Notes. 2018 Jan 24;11:68. doi: 10.1186/s13104-018-3177-0 (PMC5782382; doi:10.1186/s13104-018-3177-0)
Supplement: Supplementary file 2 — Additional file 2. Comparing OTP Outcomes with Sphere Standards. Compares various outcomes of Gado OTP with respect to Sphere standards. [file 13104_2018_3177_MOESM2_ESM.docx]

S2

| Outcome | Sphere | OTP |
| --- | --- | --- |
| Recovery Rate | >75% | 72.8% |
| Mortality Rate | <10% | 0.4% |
| Rate of Defaulting | <15% | 0% |
| RWG  (Median (IQR) | ≥5g/kg/day | 3.8 (2.1-6.6) |
